# Associations between socioeconomic status and risk of obesity and overweight among Chinese children and adolescents

**DOI:** 10.1186/s12889-023-15290-x

**Published:** 2023-02-28

**Authors:** Youzhi Ke, Shikun Zhang, Yueran Hao, Yang Liu

**Affiliations:** 1 School of Physical Education, Shanghai University of Sport, Shanghai, China; 2 Shanghai Research Center for Physical Fitness and Health of Children and Adolescents, Shanghai University of Sport, Shanghai, China

**Keywords:** Socioeconomic status, Overweight, Obesity, Body mass index, Youth

## Abstract

**Background:**

In China, the threat of obesity and overweight in children and adolescents is developing quickly. It may be possible to lower the risk of obesity and overweight in children and adolescents by understanding the factors that drive these conditions. Therefore, this study aimed to investigate the association between SES and risk of obesity and overweight among children and adolesecnts in China’s provinces of Jiangsu, Anhui, Zhejiang, and Shanghai.

**Methods:**

Chinese children and adolescents (*n* = 2,746; 46.3% boys) were recruited using multistage sampling. SES was measured using self-reported questionnaires, the specific indicators were parental education, perceived family wealth, and Family Affluence Scale II. Height and weight were measured and used to calculate body mass index (BMI, categorized into obesity or overweight). The definition of obesity or overweight was based on the Chinese standard "Screening for obesity and overweight among school-age children and adolescents". Descriptive statistics, independent sample t-tests, and a Chi-square test were used to report the sample characteristics and analyse BMI differences across different sociodemographic groups. A binary logistic regression was then applied to analyse the association of SES indicators with BMI in children and adolescents.

**Results:**

Overall, 22.5% of children and adolescents were obese or overweight. Participants with medium and high maternal education levels were 1.48 [95% CI 1.15–1.91] and 1.47 [95% CI 1.03–2.11] times more likely to be obese/overweight. Girls with medium maternal education levels were 1.70[95% CI 1.21–2.40] times more likely to be obese/overweight. For boys, no association was observed. Junior middle school students with medium maternal education levels were 1.51[95% CI 1.10–2.07] times more likely to be obese/overweight. Participants with medium or high FAS, perceived family wealth, or paternal education levels were not associated with obesity/overweight.

**Conclusions:**

The findings of this study indicated a positive association between SES and risk of overweight/obesity in girls, suggesting that maternal education level may have a substantial impact on future prevention efforts for these conditions in girls. To increase the effectiveness of interventions, longitudinal studies are necessary to better understand the causal association between SES and obesity/overweight.

## Background

Obesity (OB) or being overweight (OW) in children and adolescents has become a major global public health issue [1]. Body mass index (BMI) is commonly used to assess the prevalence of overweight and obesity. BMI is one of the most important indicators of nutrition and obesity among children and adolescents, both in theory and in practice. According to relevant research findings, BMI is not only significantly related to hyperglycemia, hypertension, and other diseases, but it also directly reflects in body fat level [2]. At the same time, high BMI in children and adolescents can affect their health status in adulthood [3]. Despite the numerous dangers of being obese or overweight, the prevalence of obesity or overweight in children and adolescents remains high. A study collected 2416 population-based studies from the world and measured the height and weight of 31 million participants aged 5–19. The results showed that from 1975 to 2016, the obesity rate of girls increased from 0.7% to 5.6%, and that of boys increased from 0.9% to 7.8% [4]. According to the China Childhood Obesity Report, just in major and medium-sized cities, the obesity rate among children has reached 4.3%, and in 2014, China had the most obese population in the world [5]. If appropriate intervention strategies are not implemented, the childhood obesity rate will increase to 6% by 2030, significantly endangering the health of children [6]. The Chinese government has implemented health education initiatives and issued dietary recommendations in an effort to combat the threat of obesity. These initiatives are intended to increase people's dietary knowledge, foster the development of healthier eating patterns, and maintain a healthy lifestyle [7]. Despite progress in preventing childhood and adolescent obesity, there is still uncertainty about the key factors that must be addressed [8].

Evidence suggests that socioeconomic status (SES) is associated with overweight and obesity in children and adolescents [9]. In adolescence and early adulthood, those who experience early disadvantage are more likely to have a higher body mass index and to be overweight or obese [10], and these correlations will influence them in midlife and into old age [11, 12]. It should be noted that these negative consequences are more pronounced and long-lasting in women, as well as in early adulthood [13–15] and midlife [11, 12]. About 70% of teenagers with obesity will become obese adults when they grow up, which emphasizes the urgency and necessity of solving this problem as soon as possible [16]. Studies on overweight or obesity in most developed countries have shown a negative association between SES and overweight or obesity in children and adolescents [17–21]. In contrast, studies in developing countries have shown that the relationship between SES and overweight or obese children and adolescents is controversial. Data from the poorest countries show a positive association between SES and obesity or overweight [18, 22–26]. However, some middle-income countries have a negative association between SES and obesity or overweight [27]. In comparison to most Western countries, China has experienced the fastest economic growth and urbanization process. In addition to affecting nutrition and lifestyle, it leads to growing inequality in SES [28]. Many researchers have investigated the association between SES and overweight or obesity in the Chinese population as a potentially significant cause of illness, but the results have been inconsistent [29–33]. According to several studies, those with lower education or higher household incomes are more likely to be overweight or obesity [29–32]. Other studies have revealed that throughout the same time period, obesity rates trended upward in all SES groups, but the upward trend was more prominent in the low SES group and somewhat downward in the high SES group [33]. According to a study, there is a negative correlation between parents' SES and their children's BMI [34]. Different SES groups and stages of industrialization may exist in different parts of China, which could account for regional variations in obesity rates.

However, this issue has received little attention in the existing research [28]. With a total area of over 358,000 square kilometers and a population of around 227 million, the Yangtze River Delta region in eastern China includes Jiangsu, Anhui, Zhejiang province, and Shanghai, which are the Yangtze River Delta regional economic integration areas. These four regions' social and economic levels differ somewhat from one another from the perspective of GDP, and to some extent, these discrepancies can be interpreted as social and economic level differences. This study investigates the association between SES and children and adolescents obesity/overweight using these four locations as examples.

## Methods

### Study design and participants

This study undertook a cross-sectional school survey, which was conducted in China’s provinces of Jiangsu, Anhui, Zhejiang, and Shanghai. The participants were 3,368 children drawn from the selected primary school (Grades 4 to 6, aged 9 to 11 years old, *n* = 399), junior middle school (Grades 7 to 9, 12 to 14 years old, *n* = 1765), and junior high school (Grades 10 to 12, 15 to 17 years old, *n* = 582), with participants thus ranging in age from 9 to17 years old. In terms of responses, 2,746 students (response rate = 81.53%) completed the self-reported questionnaire, with an average age of 13.57 ± 2.26 years.

### Procedures

The study protocol and procedure were approved by the Institutional Review Board (IRB) of Shanghai University of Sport (SUS), while further permission to conduct the study was obtained from the teachers and principals of the participating schools. The IRB of SUS agreed that verbal consent was sufficient for the conduction of this study because none of the survey items related to any personal or ethical issues. All children and adolescents involved in the study were asked to answer the self-reported questionnaire. And all participants, along with their parents or guardians, were advised that participation was completely voluntary, verbal informed consent was obtained from all parents or guardians, and positive assent was obtained from all children and adolescents before data collection. Trained research assistants implemented the survey in a prearranged manner according to a standardized administration protocol during regular school hours, and the survey was thus completed on paper in the classroom setting. Students were instructed on how to complete the survey and were provided with ample time for questions. Data from the survey were then collected and analyzed anonymously.

### Measurements

#### Dependent variables

Standing height barefoot was measured using a stable stadiometer (GMCS-SGZG3, Jian-Min, Beijing) to the nearest 0.001 m. Bodyweight with light clothes was measured using a portable scale (GMCS-YERCS3, Jian-Min, Beijing) to the nearest 0.1 kg (kg). BMI was calculated by height and body weight:

$$\mathrm{BMI }(\mathrm{kg}/{m}^{2})=\frac{\mathrm{Weight }(\mathrm{kg})}{{\left(\mathrm{Height}\right)}^{2} ({m}^{2})}$$

Due to the differences in body fat percentage between East Asians and Europeans, the definition of overweight or obesity was based on the Chinese standard "Screening for overweight and obesity among school-age children and adolescents", standard number WS/T 586–2018 [35, 36]. The process for identifying overweight or obesity in school-aged children and adolescents between the ages of 6 and 18 was described in the standard, together with the technical prerequisites for this procedure. This recommendation for screening school-age children and adolescents between the ages of 6 and 18 for overweight and obesity shall apply to all regions of China. The specific specifications were detailed in Table 1.

**Table 1:** BMI screening for overweight and obesity cut-off values for school-age children and adolescents aged 6–18 years by sex and age

Age	Boy		Girl	
	**Overweight**	**Obesity**	**Overweight**	**Obesity**
6.0 ~	16.4	17.7	16.2	17.5
6.5 ~	16.7	18.1	16.5	18.0
7.0 ~	17.0	18.7	16.8	18.5
7.5 ~	17.4	19.2	17.2	19.0
8.0 ~	17.8	19.7	17.6	19.4
8.5 ~	18.1	20.3	18.1	19.9
9.0 ~	18.5	20.8	18.5	20.4
9.5 ~	18.9	21.4	19.0	21.0
10.0 ~	19.2	21.9	19.5	21.5
10.5 ~	19.6	22.5	20.0	22.1
11.0 ~	19.9	23.0	20.5	22.7
11.5 ~	20.3	23.6	21.1	23.3
12.0 ~	20.7	24.1	21.5	23.9
12.5 ~	21.0	24.7	21.9	24.5
13.0 ~	21.4	25.2	22.2	25.0
13.5 ~	21.9	25.7	22.6	25.6
14.0 ~	22.3	26.1	22.8	25.9
14.5 ~	22.6	26.4	23.0	26.3
15.0 ~	22.9	26.6	23.2	26.6
15.5 ~	23.1	26.9	23.4	26.9
16.0 ~	23.3	27.1	23.6	27.1
16.5 ~	23.5	27.4	23.7	27.4
17.0 ~	23.7	27.6	23.8	27.6
17.5 ~	23.8	27.8	23.9	27.8
18.0 ~	24.0	28.0	24.0	28.0

Note: unit: kg / m^2^

#### Independent variables

The individual measures of SES were examined based on Family Affluence Scale II (FAS II), parental education, and perceived family wealth. The FAS II has been used extensively in the Health Behaviour in School-aged Children study in the past decade to examine and describe socioeconomic inequalities in adolescent health outcomes [37]. The 3-point FAS II (low, medium, or high) was developed based on four measures of material family wealth, as reported by the students (car ownership, bedroom sharing, holiday travel, and computer ownership) [38]. FAS II was assessed by using the question: "Do you have a car at home?" (No = 0; one = 1; yes, more than two = 2); "How many computers do you have at home?" (No = 0; one set = 1; two sets = 2; more than two sets = 3); "In the past year, how many times did you and your family travel during the holidays?" (No = 0; once = 1; twice = 2; more than twice = 3); "Do you have your own room?" (No = 0; Yes = 1). In the analysis process, FAS II was divided into three categories ("low", "medium" and "high"). The FAS II category corresponds to the tertile of the total score ("low" = 0–2; "medium" = 3–5, "High" = 6–9) [39].

Parental education was determined based on reported data, categorizing parental educational experience into seven groups: 1) Below elementary school; 2) Elementary school; 3) Junior middle school; 4) High school or occupational school; 5) College; 6) Undergraduate; and 7) Postgraduate and above. In the process of analysis, the parental educational backgrounds were divided into three categories: a low education level (below elementary school, elementary school, and junior middle school), a medium education level (high school or occupational school and college), and a high education level (undergraduate or postgraduate and above) [39].

The perceived family wealth measure was designed to assess the students’ perceptions of their family’s SES. This variable was developed from the question “How well off do you think your family is?” with the available response categories being “very well off”, “quite well off”, “average”, “not very well off”, and “not at all well off”. In the process of analysis, perceived family wealth was again divided into three categories: a low economic level (not very well off and not at all well off), a medium economic level (average), and a high economic level (very well off and quite well off) [39].

#### Control variables

The following socio-demographic control variables were used: demographic information, including sex (1 = boy, 2 = girl), age (9–17 years old) and grade (Grades 4 to 12), and ethnicity (Han or other).

### Statistical analyses

The collected questionnaires were sorted in Excel and analyzed further using IBM SPSS 24. All missing cases and abnormal values were first removed, to address the aims of this study, the variables of grade, sex, and obesity/overweight were made the focus of full statistical analysis. Descriptive statistics were used to analyze the basic situation of the survey objects (demographic information, obesity/overweight, and SES), with continuous variables expressed in the form of mean ± standard deviation, and categorical variables expressed as numbers (n) or percentages (%). Independent sample t-tests and chi-square tests were used to analyze the differences between the characteristics of the different samples. Binary logistic regression was used to analyze the association of SES and BMI, adjusted for sociodemographic factors. All logistic regression analysis results were presented as odds ratios (OR) with a 95% confidence interval (CI). All *p* ≤ 0.05 were deemed statistically significant.

## Results

The descriptive characteristics of the samples in this study were shown in Table 2. A total of 2,746 subjects were finally included in the survey, including 1,272 boys (46.3%) and 1,474 girls (53.7%), with an average age of 13.57 ± 2.26 years (13.36 ± 2.18 of boys, 13.74 ± 2.30 of girls, *p* < 0.001). The proportion of primary school was 14.5% (boys 15.0%, girls 14.1%); junior middle school accounted for 64.3% (boys 71.7%, girls 57.9%); The proportion of high school was 21.2% (boys 13.3%, girls 28.0%). There was a statistically significant sex difference among grade groups (*p* < 0.001). The BMI of the participants was 19.83 kg/m^2^ (20.15 kg/m^2^ for boys, 19.55 kg/m^2^ for girls, *p* < 0.001). The majority of participants were Han Chinese (97.2%), and no significant difference was found between ethnic groups (*p* > 0.05). 42.3% of the participants reported that their fathers had low levels of education (41.0% for boys,43.5% for girls, *p* > 0.05). And 49.4% of the participants reported that their mothers had low levels of education (47.5% for boys,51.1% for girls, *p* > 0.05). About 58.6% of the participants had perceived family wealth to be medium (56.2% for boys and 60.6% for girls), and 13.3% of participants with low FAS (13.7% for boys, 12.9% for girls, *p* > 0.05).

**Table 2:** The Characteristics of the Sample

	Overall (2746)	Boys (1272)	Girls (1474)	*P*
**Height(cm)**	159.51	161.63	157.68	< 0.001
**Weight(**kg**)**	50.92	53.25	48.97	< 0.001
**BMI(kg/m**^**2**^**)**	19.83	20.15	19.55	< 0.001
**Age (years), M ± SD**	13.57 ± 2.26	13.36 ± 2.18	13.74 ± 2.30	< 0.001
**Grade groups, n (%)**				
Primary school	399(14.5)	191(15.0)	208(14.1)	< 0.001
Junior middle school	1765(64.3)	912(71.7)	853(57.9)	
High school	582(21.2)	169(13.3)	413(28.0)	
**Ethnicity, n (%)**				
Han	2663(97.0)	1236(97.2)	1427(96.8)	0.920
Others	83(3.0)	36(2.8)	47(3.2)	
**SES, n (%)**				
**Paternal education level**				
Low	1163(42.3)	521(41.0)	642(43.5)	0.390
Medium	955(34.8)	453(35.6)	502(34.1)	
High	628(22.9)	298(23.4)	330(22.4)	
**Maternal education level**				
Low	1357(49.4)	604(47.5)	753(51.1)	0.169
Medium	832(30.3)	401(31.5)	431(29.2)	
High	557(20.3)	267(21.0)	290(19.7)	
**Perceived family wealth**				
Low	319(11.6)	139(10.9)	180(12.2)	0.005
Medium	1608(58.6)	715(56.2)	893(60.6)	
High	819(29.8)	418(32.9)	401(27.2)	
**FAS**				
Low	364(13.3)	174(13.7)	190(12.9)	0.598
Medium	1143(41.6)	517(40.6)	626(42.5)	
High	1239(45.1)	581(45.7)	658(44.6)	

*M* ± *SD* mean ± standard deviation; BMI mean body mass index

SES means socioeconomic status; FAS means Family Affluence Scale

*P* values: sex differences

Primary school: 9–11 years old

Junior middle school: 12–14 years old

High school: 15–17 years old

Table 3 shows the prevalence of obesity or overweight among participants. The percentage of OW or OB was 22.5%, and boys were higher than girls (29.8% vs 16.3%, *p* < 0.001). The percentages of OW or OB in the three grade groups were different (primary school: 36.1%; junior school: 19.7%; high school: 22.0%, *p* < 0.001).

**Table 3:** The Prevalence of OW/OB

	non-OW/OB		OW/OB		*P*
	n	%	n	%	
***Total***	2127	77.5	619	22.5	< 0.001
***Sex***					
Boy	893	70.2	379	29.8	< 0.001
Girl	1234	83.7	240	16.3	
***Grade groups***					
Primary school	255	63.9	144	36.1	< 0.001
Junior middle school	1418	80.3	347	19.7	
High school	454	78.0	128	22.0	

*OW* means Overweight, *OB* means Obesity

*P* values: Differences between non-OW/OB and OW/OB

Primary school: 9–11 years old

Junior middle school: 12–14 years old

High school: 15–17 years old

**Fig. 1:**
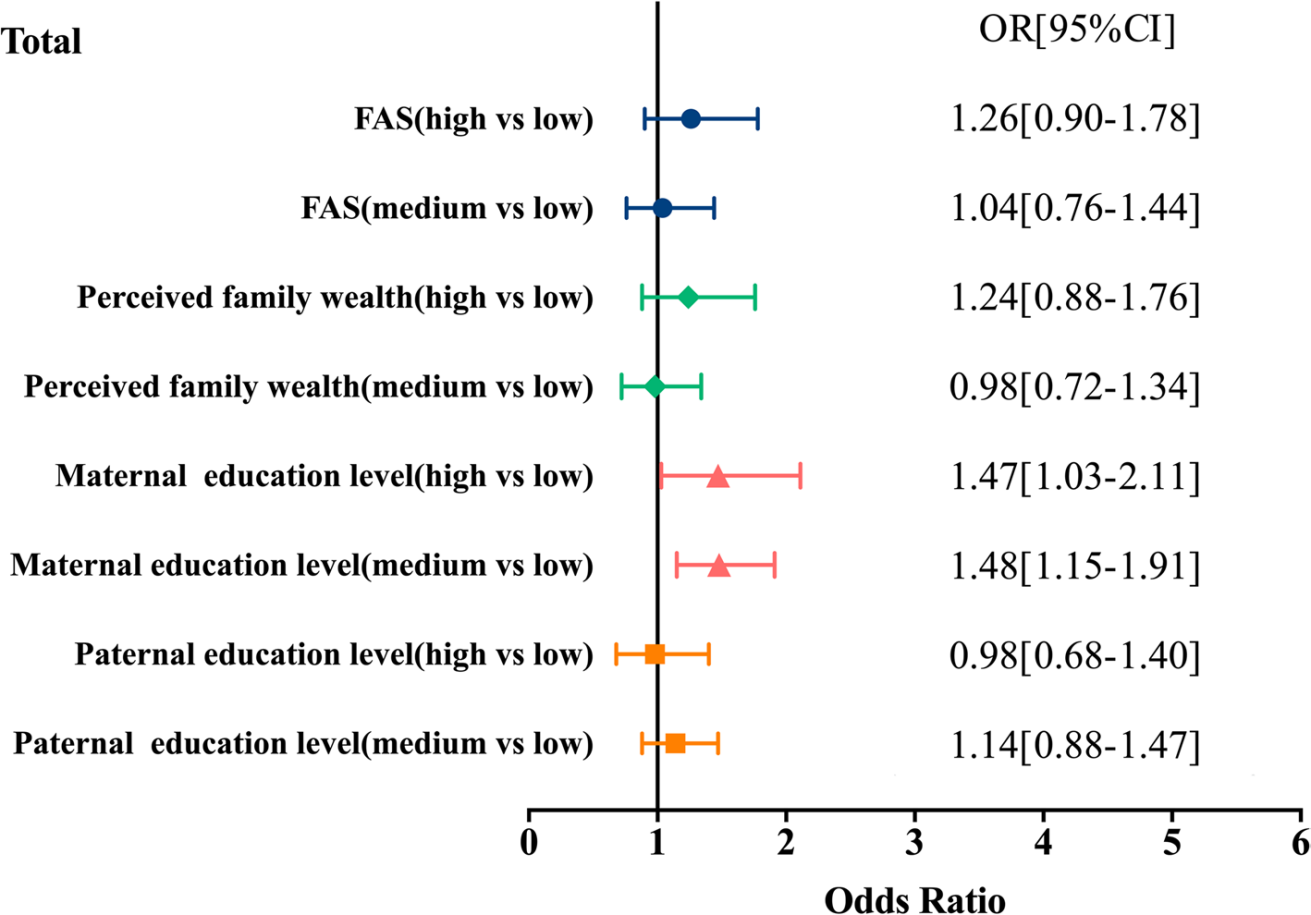
Regression analysis of the socioeconomic status and body mass index

The associations between SES and BMI in adolescents are presented in Fig. 1. Participants with medium and high maternal education levels were 1.48 [95% CI 1.15–1.91] and 1.47 [95% CI 1.03–2.11] times more likely to be obese/overweight than participants with low maternal education levels. Participants with medium or high FAS, perceived family wealth, or paternal education levels were not associated with overweight or obesity.

**Fig. 2:**
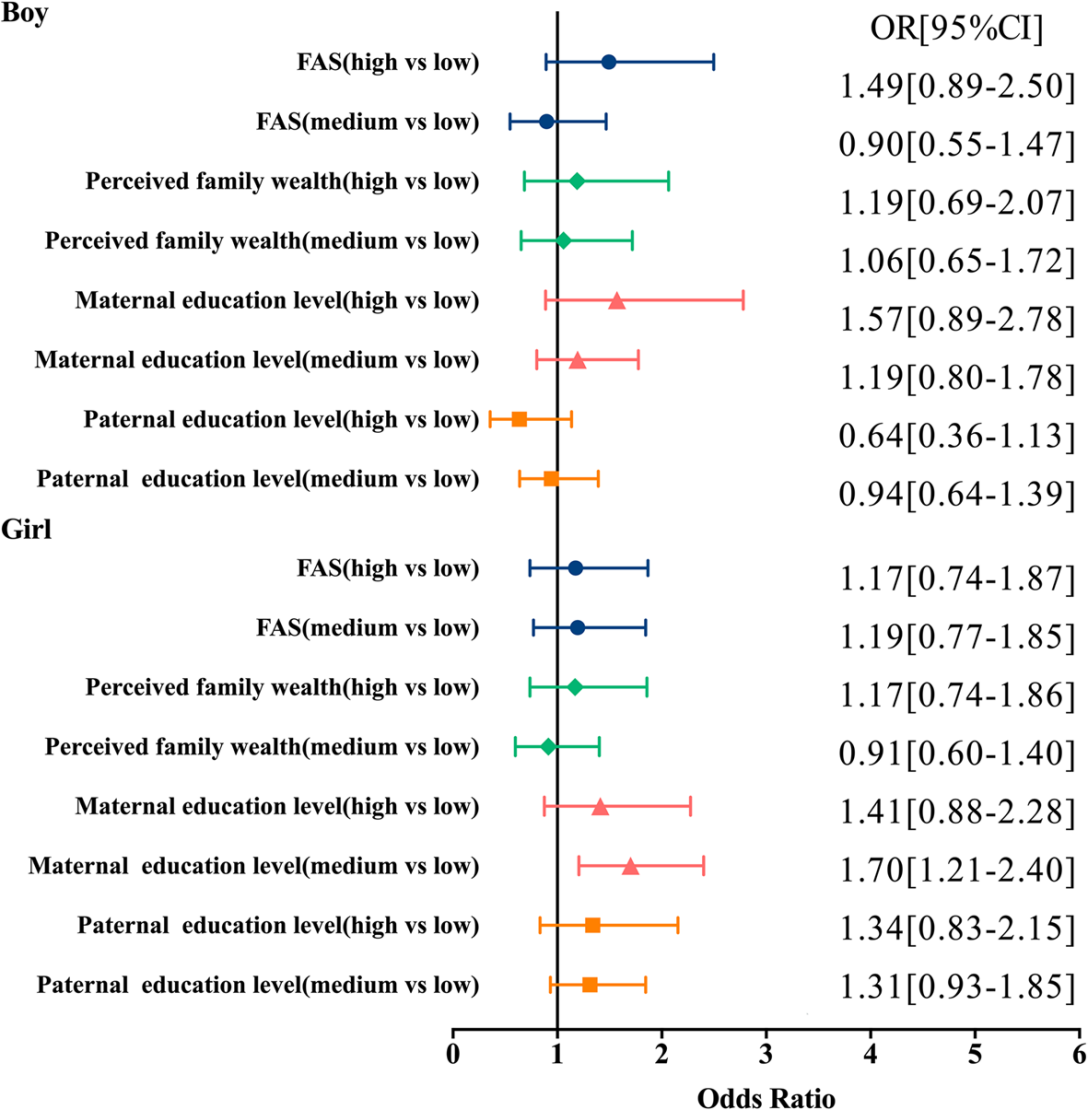
Regression analysis of sex differences in socioeconomic status and body mass index

The summarized results for the OR for participants whose BMI was overweight or obese by sex are shown in Fig. 2. Boys with medium or high FAS, perceived family wealth, and parental education levels were not associated with overweight or obesity. The findings only found that girls with medium maternal education levels were 1.70[95% CI:1.21–2.40] times more likely to be obesity/overweight than participants with low maternal education levels. Girls with medium or high FAS, perceived family wealth, or paternal education levels were not associated with the obesity/overweight.

**Fig. 3:**
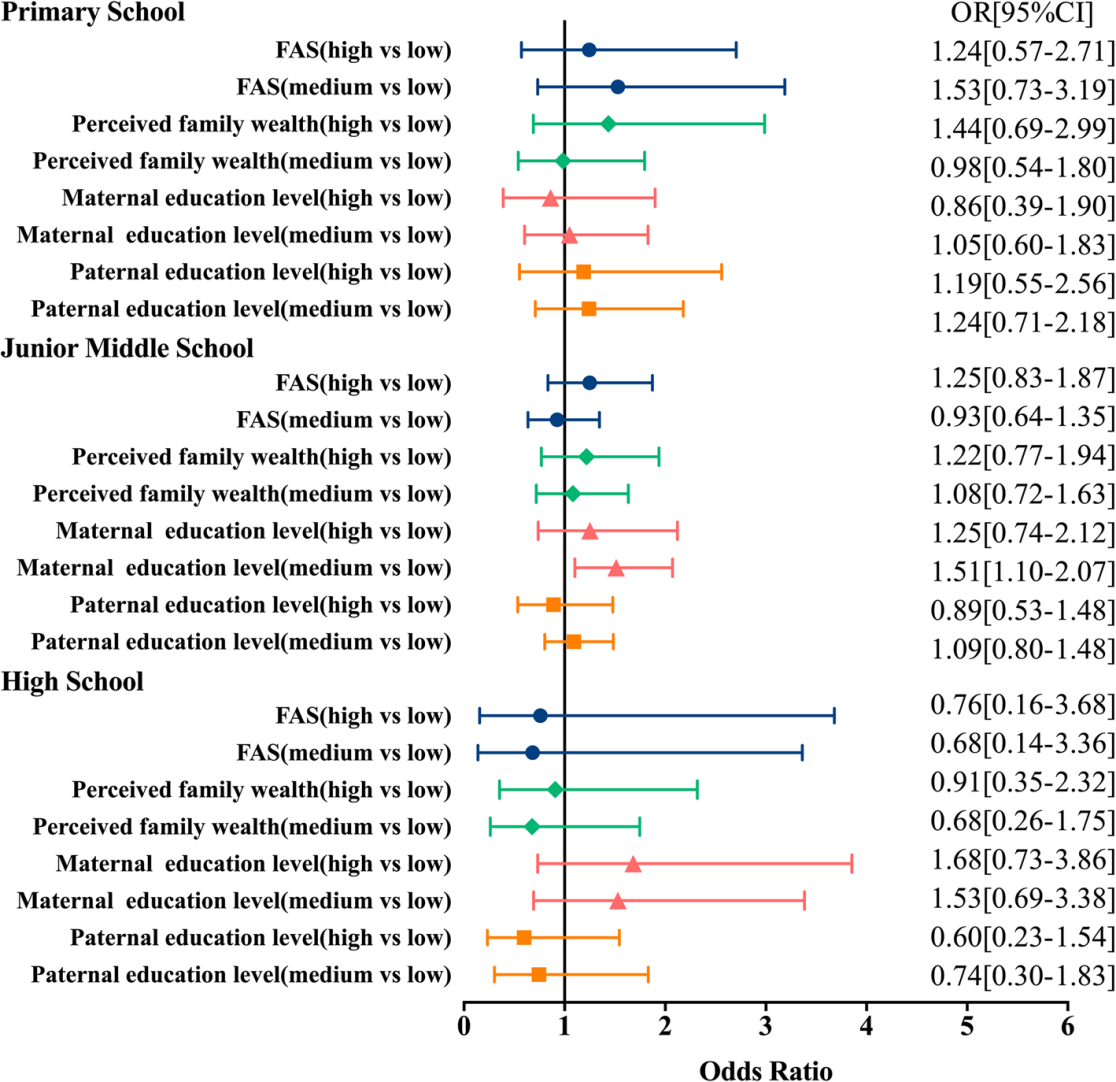
Regression analysis of grade differences in socioeconomic status and body mass index

The summarized results for the OR for participants whose BMI was overweight or obese by grade group are shown in Fig. 3. Participants from junior middle school students with medium maternal education levels were 1.51[95% CI: 1.10–2.07] times more likely to be obese/overweight than participants with low maternal education levels. Participants from primary and high school students with medium or high FAS, family wealth perceived ability, or parental education levels were not significantly associated with obesity/overweight.

## Discussion

Maternal education level is significantly correlated with obesity/overweight among children and adolescents, particularly for girls in junior middle school, according to an analysis of the association between SES and obesity or overweight in Chinese children and adolescents. There was no association between other SES indicators and obesity or overweight in children and adolescents. This demonstrates that in China, we should pay more attention to the influence of maternal education levels on overweight or obesity in children and adolescents, particularly among highly educated mothers.

The economic growth that China is experiencing so quickly makes it possible to guarantee children's and teenagers' nutrition. Compared to families from poorer socioeconomic backgrounds, mothers with higher education are more likely to provide their kids with the high-fat, high-sugar meals they prefer, which may contribute to children and adolescents being overweight and obesity [8]. However, women with less education may be busier at work, their kids may need help with chores in their free time, and they may have fewer chances of picking up undesirable behaviours (such as being sedentary and eating fast food) [40]. Other SES indicators, however, do not significantly associate with obesity or overweight in children and adolescents, suggesting that there is only a tenuous association between SES and obesity or adolescent overweight in children and adolescents. Sedentary behaviour or physical activity may have a significant impact on obesity or overweight in children and adolescents as a result of the changing social economy and way of life [6, 41].

Some developing countries, like China, have seen a growth in obesity or overweight rates more quickly in recent years than other developed countries, like the USA [42]. According to numerous studies, low-SES groups in developing countries and high-SES groups in developing countries (including China) with greater access to calorie-dense foods are more likely to acquire obesity than their counterparts [42, 43]**.** And there are also differences between rural and urban areas in China, the association between SES and overweight and obesity is higher in urban areas than in rural areas [44]. In terms of specific indicators, there are also studies from Chinese regions that differ from our results, for example, one study showed that the prevalence of overweight and obesity among children in Chongqing was positively associated with the paternal education level but not with the mother's [45]. However, a longitudinal survey of the China Family Panel Studies showed that the prevalence of overweight and obesity was only negatively associated with the parental education level up to the age of 10 years but not in children 11–15 years old [46]. Our findings conflict with earlier research, which demonstrates a significant inverse relationship between SES and BMI [47, 48]. There are two main reasons for the conflicting results. First of all, there is no universal method for classifying SES, and various SES indicators correspond to various SES latitudes. Several indicators of SES can be used to study the association between SES and BMI, and each indicator has its advantages and disadvantages [49]. For instance, it has been demonstrated that the number of family members and BMI is positively correlated, whereas the educational attainment of parents and BMI are negatively correlated [50]. The results were masked by the use of various SES indicator combinations. Studies indicate that there are no discernible differences between SES and BMI when the occupation is used to represent SES [51]. Children and adolescents find it challenging to appropriately describe the jobs of their parents in self-reported questionnaires [52]. This is another justification for not using the occupation indicator in this analysis. The household income index is a measure of social and economic standing, however, there is significant debate around it. Its sensitivity results in a poor response rate [53]. FAS is now extensively utilized as an accepted measure of children's and adolescents' SES [37]. And indicators of parental education level are frequently used to assess the legitimacy and respect of SES [54, 55]. It is very important to note that perceived family wealth is children's and adolescents' subjective assessment of their family's SES. Thus, the FAS, parental education level, and perceived family wealth are mostly used in this study as the main indicators to evaluate the SES. The evaluation standard of obesity/overweight is another potential factor. Different countries define being overweight or obese in different ways. For instance, some studies utilize the International Obesity Task Force standard and some studies use the WHO classification criteria for BMI [28, 56] The proportions of overweight or obese people vary according to different standards, which will have an effect on the findings of the study. The BMI classification released in China was chosen based on the real circumstances of the study participants.

Boys and girls are equally unaffected by parental education levels, FAS, and perceived family wealth. However, girls with medium maternal education levels were more likely to be obese/overweight. The possible causes of this could be that mothers with higher education are stricter about controlling the physical size of girls, while boys are generally more tolerant [57–59]. On the other hand, girls are more concerned about body image and more interested in weight control than boys [60]. Maybe it's because boys consume more physical strength and eat more food, and they tend to eat more calories without being aware of weight control.

This study found a significant association between higher maternal education and obesity or overweight in junior middle school students across a range of grade levels. Mothers with higher education levels are more likely to meet or satisfy their children's nutritional demands since junior middle school pupils are in puberty, which is also a crucial time for their growth. And students in junior middle school are also at a point where they can form their own opinions about the outside world and are more likely to be open to trying new things [61]. Additionally, there is more pressure on kids to continue their education during their high school years. Teenagers are more likely to enroll in various extracurricular tutoring classes if their mothers have advanced degrees [62]. As a result, more time is spent sitting down, less time is spent exercising, and more stress is felt. All of these could raise the likelihood of obesity or overweight people. Teenagers whose mothers have low levels of education, however, experience significantly fewer limitations and have more free time for extracurricular activities. As a result, the likelihood of being overweight or obese during this time is relatively low.

Future research should focus on SES-affected girls, as they are more likely to experience the risk of being overweight or obese. A cross-sectional study has revealed that teenagers with high BMI had poor physical fitness, supporting earlier findings that obesity causes speed, strength, flexibility, and other physical fitness to decline noticeably [63]. Furthermore, obesity and being overweight can lead to noncommunicable diseases such as diabetes, cancer, and musculoskeletal disorders [64, 65]. It is important to note that children and adolescents who are obese or overweight are more likely to have sedentary habits (e.g., spend more time watching TV) [66], and spend less time participating in physical activities [67], resulting in poorer physical health. Overweight or obese children and teenagers need the attention of the government, school departments, and parents.

Our study has a number of limitations. First, cross-sectional data rather than longitudinal data was used. Prospective longitudinal studies may assist identify risk factors causing obesity in Chinese children and adolescents because BMI might fluctuate over time as a result of a range of causes. Second, the respondents' involvement in this study was voluntary and convenience sampling was utilized, which to some extent impacts the sample's accuracy and makes it less representative. Thirdly, it has been shown in recent literature that genes and lifestyle factors, such as nutrition and physical activity, may have an impact on BMI. The absence of information prevented this work from studying it. Fourthly, it's important to discuss how body mass index is used. Although it is useful as a demographic indicator for identifying overweight and obesity, the fact that it is not the gold standard for measuring body composition may have some limits. Despite its limitations, this study has enriched the literature in two aspects. Firstly, the SES indicators used in this study were widely recognized. Secondly, the results of this study show that among the numerous SES indicators, only maternal education levels are related to overweight or obesity among children and adolescents. This finding is inconsistent with previous research results in developed countries [2]. According to the findings of our study, children and adolescents have a socioeconomic situation that is more problematic due to obesity or overweight when compared to developed nations. More specialized programs and regulations must be created in order to significantly enhance the health behaviours of children and adolescents.

## Conclusions

The results of this study show that girls in junior middle school students with medium maternal education levels are more likely to be overweight or obese, while paternal education levels, FAS, and perceived family wealth were not associated with the overweight or obesity of children and adolescents. These discoveries might be important for public health. When examining the health behaviours of children and adolescents with high SES, it is crucial to take into account how maternal education levels may affect their children's tendency to be overweight or obese. Additionally, longitudinal research is required to enhance the efficacy of interventions and better understand the causal association between SES and BMI. To fully and successfully enhance the health of children and adolescents, people must also be aware of the risks associated with obesity and being overweight.

## Data Availability

The raw data supporting the conclusions of this article will be made available by the authors, without undue reservation. If someone wants to obtain the data from this study should contact the corresponding author.

## Abbreviations

BMI: Body mass indexes
OW: Overweight
OB: Obesity
SES: Socioeconomic status
FAS: Family affluence scale
OR: Odds ratio
CI: Confidence interval
